# New bandwidth selection criterion for Kernel PCA: Approach to dimensionality reduction and classification problems

**DOI:** 10.1186/1471-2105-15-137

**Published:** 2014-05-10

**Authors:** Minta Thomas, Kris De Brabanter, Bart De Moor

**Affiliations:** 1 KU Leuven, Department of Electrical Engineering (ESAT), STADIUS Center for Dynamical Systems, Signal Processing and Data Analytics/iMinds Medical IT, Kasteelpark Arenberg 10, 3001 Leuven, Belgium; 2 Iowa State University, Department of Statistics & Computer Science, Ames, IA, USA

## Abstract

**Background:**

DNA microarrays are potentially powerful technology for improving diagnostic classification, treatment selection, and prognostic assessment. The use of this technology to predict cancer outcome has a history of almost a decade. Disease class predictors can be designed for known disease cases and provide diagnostic confirmation or clarify abnormal cases. The main input to this class predictors are high dimensional data with many variables and few observations. Dimensionality reduction of these features set significantly speeds up the prediction task. Feature selection and feature transformation methods are well known preprocessing steps in the field of bioinformatics. Several prediction tools are available based on these techniques.

**Results:**

Studies show that a well tuned Kernel PCA (KPCA) is an efficient preprocessing step for dimensionality reduction, but the available bandwidth selection method for KPCA was computationally expensive. In this paper, we propose a new data-driven bandwidth selection criterion for KPCA, which is related to least squares cross-validation for kernel density estimation. We propose a new prediction model with a well tuned KPCA and Least Squares Support Vector Machine (LS-SVM). We estimate the accuracy of the newly proposed model based on 9 case studies. Then, we compare its performances (in terms of test set Area Under the ROC Curve (AUC) and computational time) with other well known techniques such as whole data set + LS-SVM, PCA + LS-SVM, t-test + LS-SVM, Prediction Analysis of Microarrays (PAM) and Least Absolute Shrinkage and Selection Operator (Lasso). Finally, we assess the performance of the proposed strategy with an existing KPCA parameter tuning algorithm by means of two additional case studies.

**Conclusion:**

We propose, evaluate, and compare several mathematical/statistical techniques, which apply feature transformation/selection for subsequent classification, and consider its application in medical diagnostics. Both feature selection and feature transformation perform well on classification tasks. Due to the dynamic selection property of feature selection, it is hard to define significant features for the classifier, which predicts classes of future samples. Moreover, the proposed strategy enjoys a distinctive advantage with its relatively lesser time complexity.

## Background

Biomarker discovery and prognosis prediction are essential for improved personalized cancer treatment. Microarray technology is a significant tool for gene expression analysis and cancer diagnosis. Typically, microarray data sets are used for class discovery [1,2] and prediction [3,4]. The high dimensionality of the input feature space in comparison with the relatively small number of subjects is a widespread concern; hence some form of dimensionality reduction is often applied. Feature selection and feature transformation are two commonly used dimensionality reduction techniques. The key difference between feature selection and feature transformation is that, in the former only a subset of original features is selected while the latter is based on generation of new features.

In this genomic era, several classification and dimensionality reduction methods are available for analyzing and classifying microarray data. Prediction Analysis of Microarray (PAM) [5] is a statistical technique for class prediction from gene expression data using Nearest Shrunken Centroid (NSC). PAM identifies subsets of genes that best characterize each class. LS-SVM is a promising method for classification, because of its solid mathematical foundations which convey several salient properties that other methods hardly provide. A commonly used technique for feature selection, t-test, assumes that the feature values from two different classes follow normal distributions. Several studies, especially microarray analysis, have used t-test and LS-SVM together to improve the prediction performance by selecting key features [6,7]. The Least Absolute Shrinkage and Selection Operator (Lasso) [8] is often used for gene selection and parameter estimation in high-dimensional microarray data [9]. The Lasso shrinks some of the coefficients to zero, and extend of shrinkage is determined by the tuning parameter, often obtained from cross validation.

Inductive learning systems were successfully applied in a number of medical domains, e.g. in localization of primary tumors, prognostic of recurring breast cancer, diagnosis of thyroid diseases, and rheumatology [10]. An induction algorithm is used to learn a classifier, which maps the space of feature values into the set of class values. This classifier is later used to classify new instances, with the unknown classifications (class labels). Researchers and practitioners realize that the effective use of these inductive learning systems requires data preprocessing, before a learning algorithm could be applied [11]. Due to the instability of feature selection techniques, it might be difficult or even impossible to remove irrelevant and/or redundant features from a data set. Feature transformation techniques, such as KPCA, discover a new feature space having fewer dimensions through a functional mapping, while keeping as much information, as possible in the data set.

KPCA, which is a generalization of PCA, a nonlinear dimensionality reduction technique that has proven to be a powerful pre-processing step for classification algorithms. It has been studied intensively in the last several years in the field of machine learning and has claimed success in many applications [12]. An algorithm for classification using KPCA was developed by Liu *et al.*[13]. KPCA was proposed by Schölkopf and Smola [14], by mapping features sets to a high-dimensional feature space (possibly infinite) and applying Mercer’s theorem. Suykens *et al.*[15,16] proposed a simple and straightforward primal-dual support vector machine formulation to the PCA problem.

To perform KPCA, the user first transforms the input data *x* from the original input space *F*_0_ into a higher-dimensional feature space *F*_1_ with a nonlinear transform *x*→*Φ*(*x*) where *Φ* is a nonlinear function. Then a kernel matrix *K* is formed using the inner products of new feature vectors. Finally, a PCA is performed on the centralized *K*, which is an estimate of the covariance matrix of the new feature vectors in *F*_1_. One of the commonly used kernel function is radial basis function (RBF) kernel: $K(x_{i},x_{j})=\exp\left(-\frac{∥x_{i}-x_{j}{∥}^{2}}{2h^{2}}\right)$ (RBF kernel with bandwidth *h*). Traditionally the optimal parameters (bandwidth and number of principal components) of RBF kernel function are selected in a trial and error fashion.

Pochet *et al.*[17] proposed an optimization algorithm for KPCA with RBF kernel followed by Fisher Discriminant Analysis (FDA) to find the parameters of KPCA. In this case, the parameter selection is coupled with the corresponding classifier. This means that the performance of the final procedure depends on the chosen classifier. Such a procedure could produce possible inaccurate results in the case of weak classifiers. In addition, this appears to be a time consuming procedure, while tuning the parameters of KPCA.

Most classification methods have inherent problem with high dimensionality of microarray data and hence require dimensionality reduction. The ultimate goal of our work is to design a powerful preprocessing step, decoupled from the classification method, for large dimensional data sets. In this paper, initially we explain an SVM approach to PCA and LS-SVM approach to KPCA. Next, by following the idea of least squares cross-validation in kernel density estimation, we propose a new data-driven bandwidth selection criterion for KPCA. The tuned LS-SVM formulation to KPCA is applied to several data sets and serves as a dimensionality reduction technique for a final classification task. In addition, we compared the proposed strategy with an existing optimization algorithm for KPCA, as well as with other preprocessing steps. Finally, for the sake of comparison, we applied LS-SVM on whole data sets, PCA+LS-SVM, t-test + LS-SVM, PAM and Lasso. Randomization on all data sets are carried out in order to get a more reliable idea of the expected performance.

## Data sets

In our analysis, we collected 11 publicly available binary class data sets (diseased vs. normal). The data sets are: colon cancer data [18,19], breast cancer data [20], pancreatic cancer premalignant data [21,22], cervical cancer data [23], acute myeloid leukemia data[24], ovarian cancer data [21], head & neck squamous cell carcinoma data [25], early-early stage duchenne muscular dystrophy (EDMD) data [26], HIV encephalitis data [27], high grade glioma data [28], and breast cancer data [29]. In breast cancer data [29] and high grade glioma data, all data samples have already been assigned to a training set or test set. The breast cancer data in [29] contains missing values; those values have been imputed based on the nearest neighbor method.

An overview of the characteristics of all the data sets can be found in Table 1. In all the cases, 2/3rd of the data samples of each class are assigned randomly to the training and the rest to the test set. These randomizations are the same for all numerical experiments on all data sets. This split was performed stratified to ensure that the relative proportion of outcomes sampled in both training and test set was similar to the original proportion in the full data set. In all these cases, the data were standardized to zero mean and unit variance.

**Table 1:** Summary of the 11 binary disease data sets

**Data set**	**#Samples**		**#Genes**
	**Class 1**	**Class 2**	
1: Colon	22	40	2000
2: Breast cancer I	34	99	5970
3: Pancreatic	50	50	15154
4: Cervical	8	24	10692
5: Leukemia	26	38	22283
6: Ovarian	91	162	15154
7: Head & neck squamous			
cell carcinoma	22	22	12625
8: Duchenne muscular dystrophy	23	14	22283
9: HIV encephalitis	16	12	12625
10: High grade glioma	29	21	12625
11: Breast cancer II	19	78	24188

## Methods

The methods used to set up the case studies can be subdivided into two categories: dimensionality reduction using the proposed criterion and subsequent classification.

### SVM formulation to linear PCA

Given training set${\left\{x_{i}\right\}}_{i=1}^{N}$, $x_{i}\in {\mathbb{R}}^{d}$ (*d* - dimensional data) and *N* given data points for which one aims at finding projected variables *v*^*T*^*x*_*i*_ with maximal variance. SVM formulation to PCA problem is given in [30] as follows:

$$\underset{v}{\max}\overset{N}{\underset{i=1}{\sum }}{\left[0-v^{T}x_{i}\right]}^{2}$$

where zero is considered as a single target value. This interpretation of the problem leads to the following primal optimization problem

$$\underset{v,e}{\max}J_{P}(v,e)=\gamma \frac{1}{2}\overset{N}{\underset{i=1}{\sum }}\overset{2}{\underset{i}{e}}-\frac{1}{2}v^{T}v$$

such that

$$e_{i}=v^{T}x_{i},i=1,\ldots ,\mathrm{N.}$$

This formulation states that one considers the difference between *v*^*T*^*x*_*i*_ (the projected data points to the target space) and the value 0 as error variables. The projected variables correspond to what one calls the score variables. These error variables are maximized for the given *N* data points while keeping the norm of *v* small by the regularization term. The value *γ* is a positive real constant. The Lagrangian becomes

$$ℒ(v,e;\alpha )=\gamma \frac{1}{2}\overset{N}{\underset{k=1}{\sum }}e_{k}^{2}-\frac{1}{2}v^{T}v-\overset{N}{\underset{k=1}{\sum }}\alpha_{k}\left(e_{k}-v^{T}x_{k}\right)$$

with conditions for optimality

$$\left\{\begin{array}{l} \frac{\partial ℒ}{\mathrm{\partial v}}=0\to v=\overset{N}{\underset{k=1}{\sum }}\alpha_{k}x_{k}\\ \frac{\partial ℒ}{\partial e_{k}}=0\to \alpha_{k}=\gamma e_{k}\;k=1,\ldots ,N\\ \frac{\partial ℒ}{\partial \alpha_{k}}=0\to e_{k}-v^{T}x_{k},\;k=1,\ldots ,\mathrm{N.} \end{array}\right)$$

By elimination of the variables *e*, *v* one obtains the following symmetric eigenvalue problem:

$$\left[\begin{array}{lll} x_{1}^{T}x_{1} & \ldots & x_{1}^{T}x_{N}\\ \vdots & \vdots \\ x_{N}^{T}x_{1} & \ldots & x_{N}^{T}x_{N} \end{array}\right]\left[\begin{array}{l} \alpha_{1}\\ \vdots \\ \alpha_{N} \end{array}\right]=\lambda \left[\begin{array}{l} \alpha_{1}\\ \vdots \\ \alpha_{N} \end{array}\right]$$

The vector of dual variables *α*=[*α*_1_;…;*α*_*N*_] is an eigenvector of the Gram matrix and $\lambda =\frac{1}{\gamma }$ is the corresponding eigenvalue. The score variable, $z_{n_{\text{pca}}}(x)$ of sample *x* on nth eigenvector *α*^*n*^ becomes

(1) $$z_{n_{\text{pca}}}(x)=v^{T}x=\Sigma_{i=1}^{N}\alpha_{i}^{\left(n\right)}x_{i}^{T}x$$

### LS-SVM approach to KPCA

The PCA analysis problem is interpreted as a one-class modeling problem with a target value equal to zero around which the variance is maximized. This results into a sum of squared error cost function with regularization. The score variables are taken as additional error variables. We now follow the usual SVM methodology of mapping the *d*-dimensional data from the input space to a high-dimensional feature space $\phi :{\mathbb{R}}^{d}\to {\mathbb{R}}^{n_{h}}$, where *n*_*h*_ can be infinite, and apply Mercer’s theorem [31].

Our objective is the following

(2) $$\underset{v}{\max}\overset{N}{\underset{k=1}{\sum }}{\left[0-v^{T}(\phi (x_{k})-{\hat{\mu }}_{\phi }))\right]}^{2}$$

with ${\hat{\mu }}_{\phi }=(1/N)\overset{N}{\underset{k=1}{\sum }}\phi (x_{k})$ and *v* is the eigenvector in the primal space with maximum variance. This formulation states that one considers the difference between $v^{T}(\phi (x_{k})-{\hat{\mu }}_{\phi })$ (the projected data points to the target space) and the value 0 as error variables. The projected variables correspond to what is called *score* variables. These error variables are maximized for the given *N* data points. Next, by adding a regularization term we also want to keep the norm of *v* small. The following optimization problem is formulated now in the primal weight space

(3) $$\underset{v,e}{\max}J_{P}(v,e)=\gamma \frac{1}{2}\overset{N}{\underset{k=1}{\sum }}e_{k}^{2}-\frac{1}{2}v^{T}v$$

such that

$$e_{k}=v^{T}(\phi (x_{k})-\mu_{\phi }),k=1,\ldots ,\mathrm{N.}$$

The Lagrangian yields

$$\;ℒ(v,e;\alpha )\;=\;\gamma \frac{1}{2}\overset{N}{\underset{k=1}{\sum }}e_{k}^{2}\;-\;\frac{1}{2}v^{T}v\;-\;\overset{N}{\underset{k=1}{\sum }}\alpha_{k}\left(e_{k}\;-\;v^{T}\left(\phi \left(x_{k}\right)\;-\;{\hat{\mu }}_{\phi }\right)\right)$$

with conditions for optimality

$$\;\left\{\begin{array}{l} \frac{\partial ℒ}{\mathrm{\partial v}}=0\to v=\overset{N}{\underset{k=1}{\sum }}\alpha_{k}\left(\phi \left(x_{k}\right)-{\hat{\mu }}_{\phi }\right)\\ \frac{\partial ℒ}{\partial e_{k}}=0\to \alpha_{k}=\gamma e_{k}\;k=1,\ldots ,N\\ \frac{\partial ℒ}{\partial \alpha_{k}}=0\to e_{k}-v^{T}\left(\phi \left(x_{k}\right)-{\hat{\mu }}_{\phi }\right)=0,\;k=1,\ldots ,\mathrm{N.} \end{array}\right)$$

By elimination of variables *e* and *v*, one obtains

$$\;\frac{1}{\gamma }\alpha_{k}-\overset{N}{\underset{l=1}{\sum }}\alpha_{l}{\left(\phi \left(x_{l}\right)\;-\;{\hat{\mu }}_{\phi }\right)}^{T}\left(\phi \left(x_{k}\right)\;-\;{\hat{\mu }}_{\phi }\right)\;=0\;\;\;k\;=\;1,\;\ldots \;,\mathrm{N.}$$

Defining $\lambda =\frac{1}{\gamma }$, one obtains the following dual problem

$$\Omega_{c}\alpha =\lambda \alpha$$

where Ω _*c*_ denotes the centered kernel matrix with *ij*th entry:$\Omega_{c,i,j}=K(x_{i},x_{j})-\frac{1}{N}\overset{N}{\underset{r=1}{\sum }}K(x_{i},x_{r})-\frac{1}{N}\overset{N}{\underset{r=1}{\sum }}K(x_{j},x_{r})+\frac{1}{N^{2}}\overset{N}{\underset{r=1}{\sum }}\overset{N}{\underset{s=1}{\sum }}K(x_{r},x_{s}).$

### Data-driven bandwidth selection for KPCA

Model selection is a prominent issue in all learning tasks, especially in KPCA. Since KPCA is an unsupervised technique, formulating a data-driven bandwidth selection criterion is not trivial. Until now, no such data-driven criterion was available to tune the bandwidth (*h*) and number of components (*k*) for KPCA. Typically these parameters are selected by trial and error. Analogue to least squares cross validation [32,33] in kernel density estimation, we propose a new data driven selection criterion for KPCA. Let

$$z_{n}(x)=\Sigma_{i=1}^{N}\alpha_{i}^{\left(n\right)}K(x_{i},x)$$

where $K(x_{i},x_{j})=\exp\left(-\frac{∥x_{i}-x_{j}{∥}^{2}}{2h^{2}}\right)$ (RBF kernel with bandwidth *h*) and set the target equal to 0 and denote by *z*_*n*_(*x*) the score variable of sample *x* on *n*^*t* *h*^ eigenvector *α*^(*n*)^. Here, the score variables are expressed in terms of kernel expressions in which every training point contributes. These expansions are typically dense (nonsparse). In Equation 3, the KPCA uses *L*_2_ lose function. Here we have chosen the *L*_1_ loss function to induce sparsness in KPCA. By extending the formulation in Equation 3 to *L*_1_ loss function, the following problem is formulated for kernel PCA [34].

$$\underset{v,e}{\max}J_{P}(v,e)=\gamma \frac{1}{2}\overset{N}{\underset{k=1}{\sum }}L_{1}(e_{k})-\frac{1}{2}v^{T}v$$

such that

$$e_{k}=v^{T}(\phi (x_{k})-\mu_{\phi }),k=1,\ldots ,\mathrm{N.}$$

**Figure 1:**
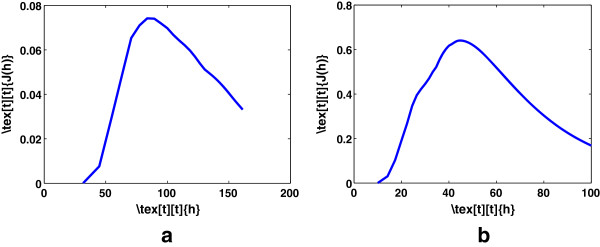
**Bandwidth selection of KPCA for a fixed number of components.** Retaining **(a)** 5 components for cervical cancer data set **(b)** 15 components for colon cancer data set.

**Figure 2:**
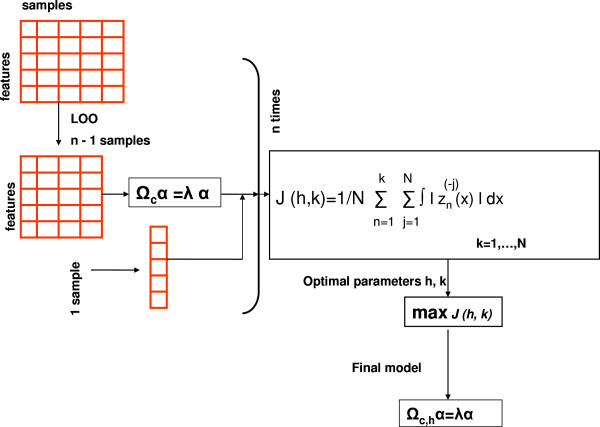
Data-Driven Bandwidth Selection for KPCA Leave-one-out cross validation (LOOCV) for KPCA.

**Figure 3:**
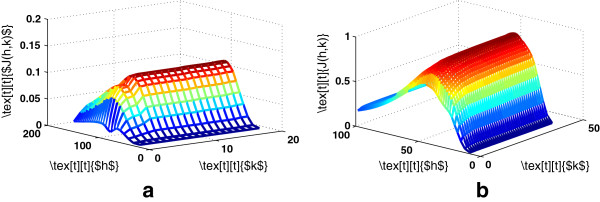
**Model selection for KPCA-optimal bandwidth and number of components.(a)** Cervical cancer **(b)** Colon cancer.

We propose the following tuning criterion for the bandwidth *h* which maximizes the *L*_1_ loss function of KPCA:

(4) $$J(h)=\underset{h\in {\mathbb{R}}_{0}^{+}}{\mathrm{argmax}}E\int |z_{n}(x)|\text{dx},$$

where *E* denotes the expectation operator. Maximizing Eq. 4 would lead to overfitting since we used all the training data in the criterion. Instead, we work with Leave-One-Out cross validation (LOOCV) estimation of *z*_*n*_(*x*) to obtain the optimum bandwidth *h* of KPCA, which gives projected variables with maximal variance. A finite approximation to Eq. 4 is given by

(5) $$J(h)=\underset{h\in {\mathbb{R}}_{0}^{+}}{\mathrm{argmax}}\frac{1}{N}\overset{N}{\underset{j=1}{\sum }}\int |\overset{\left(-j\right)}{\underset{n}{z}}(x)|\text{dx}$$

where *N* is the number of samples and $z_{n}^{\left(-j\right)}$ denotes the score variable with the *j*th observation is left out. In case the leave-one-out approach is computationally expensive, one could replace it with a leave *v* group out strategy (*v*- fold cross-validation). Integration can be performed by means of any numerical technique. In our case, we have used trapezoidal rule. The final model with optimum bandwidth is constructed as follows:

$$\Omega_{c,{ĥ}_{\text{max}}}\alpha =\lambda \alpha ,$$

where ${ĥ}_{\text{max}}=\underset{h\in {\mathbb{R}}_{0}^{+}}{\max}\frac{1}{N}\overset{N}{\underset{j=1}{\sum }}\int |\overset{\left(-j\right)}{\underset{n}{z}}(x)|\mathrm{dx.}$ Figure 1 shows the bandwidth selection for cervical and colon cancer data sets for fixed number of components. To also retain the optimum number of components of KPCA, we modify Eq. 5 as follows:

(6) $$J(h,k)=\underset{h\in {\mathbb{R}}_{0}^{+},k\in {\mathbb{N}}_{0}}{\mathrm{argmax}}\frac{1}{N}\overset{k}{\underset{n=1}{\sum }}\overset{N}{\underset{j=1}{\sum }}\int |\overset{\left(-j\right)}{\underset{n}{z}}(x)|\text{dx}$$

where *k*=1,…,*N*. Figure 2 illustrate the proposed model. Figure 3 shows the surface plot of Eq. 6 for various values of *h* and *k*.

**Figure 4:**
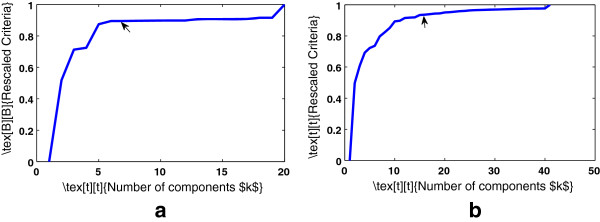
**Slice plot for the Model selection for KPCA for the optimal bandwidth.(a)** Cervical cancer **(b)** Colon cancer.

Thus, the proposed data-driven model can obtain the optimal bandwidth for KPCA, while retaining minimum number of eigenvectors which capture the majority of the variance of the data. Figure 4 shows a slice of the surface plots. The values of the proposed criterion were re-scaled to be maximum 1. The parameters that maximize Eq. 6 are *h*=70.71 and *k*=5 for cervical cancer data and *h*=43.59 and *k*=15 for colon cancer data.

### Classification models

The constrained optimization problem for an LS-SVM [16,35] for classification has the following form:

$$\underset{w,b,e}{\min}\left(\frac{1}{2}w^{T}w+\gamma \frac{1}{2}\Sigma_{k=1}^{N}e_{k}^{2}\right)$$

subject to:

$$y_{k}\left[w^{T}\phi (x_{k})+b\right]=1-e_{k},\;k=1,\ldots ,N$$

where *ϕ*(.): ${\mathbb{R}}^{d}\to {\mathbb{R}}^{d_{h}}$ is a nonlinear function which maps the *d*-dimensional input vector *x* from the input space to the *d*_*h*_-dimensional feature space, possibly infinite. In the dual space the solution is given by

$$\left[\begin{array}{ll} 0 & y^{T}\\ y & \Omega +\frac{I}{\gamma } \end{array}\right]\left[\begin{array}{l} b\\ \beta \end{array}\right]=\left[\begin{array}{l} 0\\ 1_{v} \end{array}\right]$$

with *y*=[ *y*_1_,…,*y*_*N*_]^*T*^,1_*N*_=[ 1,…,1]^*T*^,*e*=[ *e*_1_,…,*e*_*N*_]^*T*^, *β*=[ *β*_1_,…,*β*_*N*_]^*T*^ and Ω _*i*,*j*_=*y*_*i*_*y*_*j*_*K*(*x*_*i*_,*x*_*j*_) where *K*(*x*_*i*_,*x*_*j*_) is the kernel function. The classifier in the dual space takes the form

(7) $$y(x)=\text{sign}\left[\overset{N}{\underset{k=1}{\sum }}\beta_{k}y_{k}K(x,x_{k})+b\right]$$

where *β*_*k*_ are Lagrange multipliers.

## Results

First we considered nine data sets described in Table 1. We have chosen the RBF kernel $K(x_{i},x_{j})=\exp\left(-\frac{||x_{i}-x_{j}|{|}^{2}}{2h^{2}}\right)$ for KPCA. In this section all the steps are implemented using Matlab R2012b and LS-SVMlab v1.8 toolbox [36]. Next, we compared the performance of the proposed method with classical PCA and an existing tuning algorithm for RBF-KPCA developed by Pochet *et al.*[17]. Later, with the intention to comprehensively compare PCA+LS-SVM and KPCA+LS-SVM with other classification methods, we applied four widely used classifiers to the microarray data, being LS-SVM on whole data sets, t-test + LS-SVM, PAM and Lasso. To fairly compare kernel functions of the LS-SVM classifier; linear, RBF and polynomial kernel functions are used (in Table 2 referred to as linear/poly/RBF). The average test accuracies and execution time for all these methods when applied to the 9 case studies are shown in Table 2 and Table 3 respectively. Statistical significance test results (two-sided signed rank test) are given in Table 4 which compares the performance of KPCA with other classifiers. For all these methods, training on 2/3rd of the samples and testing on 1/3rd of the samples was repeated 30 times.

**Table 2:** Comparison of classifiers: Mean AUC(std) of 30 iterations

**Data set**	**Kernel function**		**Preprocessing +**	**LS-SVM classifier**		**PAM**	**Lasso**
	**for classification**						
		**Whole data**	**PCA**	**KPCA**	**t-test ( *p *** **<0 *. *** **05)**		
	RBF	0.769(0.127)	0.793(0.081)	0.822(0.088)	0.835(0.078)		
I	lin	0.822(0.068)	0.837(0.088)	**0.864**(0.078)	**0.857**(0.078)	0.787(0.097)	**0.837**(0.116)
	poly	0.818(0.071)	0.732(0.072)	0.825(0.125)	0.845(0.017)		
	RBF	0.637(0.146)	0.749(0.093)	**0.780**(0.076)	0.779(0.082)		
II	lin	**0.803**(0.059)	0.772(0.094)	**0.790**(0.075)	0.751(0.071)	0.659(0.084)	0.766(0.074)
	poly	0.701(086)	0.752(0.063)	0.753(0.072)	0.784(0.059)		
	RBF	0.832(0.143)	0.762(0.066)	0.879(0.058)	0.921(0.027)		
III	lin	**0.915**(0.043)	0.785(0.063)	0.878(0.066)	**0.941**(0.036)	0.707(0.067)	**0.9359**(0.0374)
	poly	0.775(0.080)	0.685(0.105)	0.8380(0.068)	0.858(0.042)		
	RBF	0.615(0.197)	0.853(0.112)	0.867(0.098)	0.808(0.225)		
IV	lin	**0.953**(0.070)	0.917(0.083)	**0.929**(0.077)	**0.987**(0.028)	0.759(0.152)	0.707(0.194)
	poly	0.762(0.118)	0.811(0.140)	0.840(0.131)	0.779(0.123)		
	RBF	0.807(0.238)	0.790(0.140)	0.976(0.035)	0.998(0.005)		
V	lin	**0.997**(0.005)	0.528(0.134)	0.982(0.022)	**0.998**(0.006)	0.923(0.062)	0.934(0.084)
	poly	0.942(0.051)	0.804(0.121)	0.975(0.028)	**0.965**(0.049)		
	RBF	**0.998**(0.001)	0.982(0.002)	0.984(0.012)	**0.998**(0.004)		
VI	lin	0.990(0.005)	0.973(0.002)	0.978(0.013)	0.993(0.013)	0.960(0.016)	0.951(0.045)
	poly	**0.998**(0.006)	0.985(0.016)	0.973(0.018)	0.995(0.011)		
	RBF	0.946(0.098)	0.941(0.057)	0.932(0.071)	0.967(0.048)		
VII	lin	**0.983**(0.025)	0.947(0.047)	**0.954**(0.051)	**0.987**(0.022)	0.931(0.058)	0.952(0.030)
	poly	0.785(0.143)	0.903(0.078)	0.915(0.080)	0.920(0.025)		
	RBF	0.823(0.159)	0.923(0.096)	0.858(0.113)	0.950(0.150)		
VIII	lin	0.840(0.164)	0.969(0.044)	0.800(0.019)	**0.999**(0.005)	**0.982**(0.050)	0.890(0.081)
	poly	0.781(0.186)	0.870(0.117)	0.785(0.121)	**0.998**(0.007)		
	RBF	0.638(0.210)	0.823(0.159)	0.852(0.180)	0.815(0.200)		
IX	lin	**0.931**(0.126)	0.840(0.164)	0.846(0.143)	**0.930**(0.139)	0.703(0.175)	0.705(0.174)
	poly	0.841(0.176)	0.781(0.186)	0.798(0.193)	0.768(0.193)		

p-value: False Discovery Rate (FDR) corrected.

**Table 3:** Summary of averaged execution time of classifiers over 30 iterations in seconds

**Data set**	**Whole data**	**PCA**	**KPCA**	**t-test ( *p *** **<0 *. *** **05)**	**PAM**	**Lasso**
1: Colon	17	10	18	13	8	72
2: Breast	56	38	54	42	12	258
3: Pancreatic	17	12	26	19	20	453
4: Cervical	43	28	29	33	43	106
5: Leukemia	225	185	184	195	28	680
6: Ovarian	51	25	39	44	19	865
7: Head & neck squamous						
cell carcinoma	59	39	45	47	30	238
8: Duchenne muscular dystrophy	146	115	113	110	80	20100
9: HIV encephalitis	45	27	27	28	88	118

**Table 4:** Statistical significance test which compares KPCA with other classifiers: whole data, PCA, t-test, PAM and Lasso

**Kernel function**	**Data set**	**I**	**II**	**III**	**IV**	**V**	**VI**	**VII**	**VIII**	**IX**
	Whole data	1.0000	1.0000	0.9250	**0.0015**	0.5750	**0.0400**	**0.0628**	**0.0200**	**0.0150**
	PCA	**0.0050**	**0.0021**	**0.0003**	**0.0015**	**2.83E-08**	**5.00E-07**	**0.0250**	**0.0005**	**0.0140**
RBF	t-test	1.0000	1.0000	1.0000	1.0000	**6.50E-04**	**4.35E-04**	**0.0110**	**0.0005**	1.0000
	PAM	1.0000	**6.10E-05**	**0.0002**	0.0800	0.1450	**0.0462**	1.0000	**0.0002**	**0.0015**
	Lasso	**0.0278**	1.000	**0.0001**	**0.0498**	1.0000	**0.0015**	1.0000	**0.00003**	**0.0200**
	Whole data	1.0000	0.3095	1.0000	1.0000	1.0000	1.0000	1.0000	**0.0009**	1.0000
	PCA	**7.00E-05**	**0.0011**	**1.30E-09**	**7.70E-09**	**1.28E-08**	**2.72E-05**	**6.15E-07**	0.357	0.230
lin	t-test	1.0000	0.2150	0.7200	1.0000	**0.0559**	**0.0443**	1.0000	0.5450	1.0000
	PAM	**0.0400**	**0.0003**	**0.0422**	**0.0015**	**0.0004**	**0.0001**	**0.0015**	1.0000	**0.0300**
	Lasso	0.4950	0.4950	**0.0049**	**2.12E-06**	**0.0005**	**0.0493**	**0.0025**	1.0000	**2.12E-06**
	Whole data	1.0000	**0.0100**	1.0000	**4.16E-11**	**0.00450**	**5.90E-08**	**7.70E-08**	1.0000	1.0000
	PCA	**0.0130**	**0.0003**	**4.35E-07**	**4.50E-05**	**7.70E-08**	**0.0040**	**3.28E-08**	**2.72E-05**	**5.00E-11**
poly	t-test	1.0000	1.0000	**0.0250**	1.0000	**0.0443**	0.2100	1.0000	**0.0005**	1.0000
	PAM	0.1200	**0.0005**	**0.0100**	**0.0400**	**0.0300**	1.0000	**0.0015**	**0.0200**	0.0650
	Lasso	**0.0100**	1.0000	**4.61E-05**	**1.76E-08**	0.5000	1.0000	**0.0006**	**0.0010**	0.4350

P-values of two-sided signed test are given.

p-value: False Discovery Rate (FDR) corrected.

### Comparison between the proposed criterion and PCA

For each data set, the proposed methodology is applied. This methodology consists of two steps. First, Eq. 6 is maximized in order to obtain an optimal bandwidth *h* and corresponding number of components *k*. Second, the reduced data set is used to perform a classification task with LS-SVM. We retained 5 and 15 components respectively for cervical and colon cancer data sets. For PCA, the optimal number of components were selected by slightly modifying the Equation 6, i.e., which performed only for the components *k* as follows:

(8) $$J(k)=\underset{k\in {\mathbb{N}}_{0}}{\mathrm{argmax}}\frac{1}{N}\overset{k}{\underset{n=1}{\sum }}\overset{N}{\underset{j=1}{\sum }}\int \left|\overset{\left(-j\right)}{\underset{n_{\text{pca}}}{z}}(x)\right|\text{dx}$$

where $z_{n_{\text{pca}}}(x)$ the score corresponding to the varibale *x* on PCA problem. (See Equation 1).

**Figure 5:**
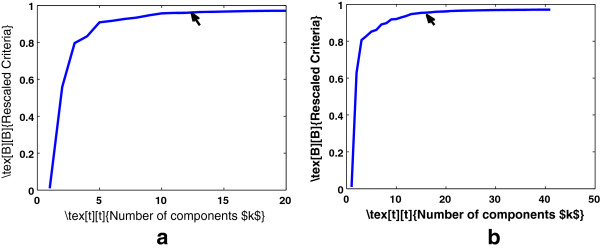
**Plot for the selection of optimal number of components for PCA.(a)** Cervical cancer **(b)** Colon cancer.

Figure 5 shows the plots of the optimal components selection of PCA. Thus we retained 13 components and 15 components for cervical and colon cancer respectively for PCA. Similarly, we obtained number of components of PCA and the number of components with corresponding bandwidth for KPCA for the remaining data sets.

The score variables (projection of samples onto the direction of selected principal components) are used to develop an LS-SVM classification model. The averaged test AUC values over the 30 random repetitions were reported.

**Figure 6:**
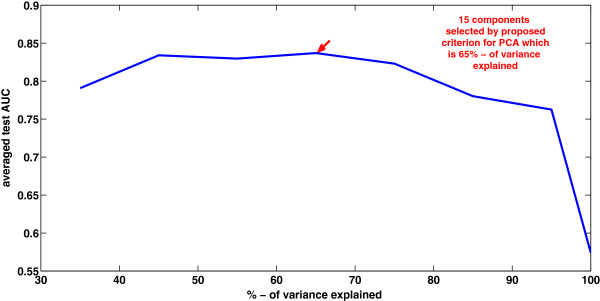
**The prediction performances on colon cancer data, with PCA+LS-SVM(RBF).** Number of selected components depends on the chosen fraction of explained total variance.

The main goal of PCA is the reduction of dimensionality, that is, focusing on a few principal components (PC) versus many variables. There are several criteria have been proposed for determining how many PC should be investigated and how many should be ignored. One common criteria is to include all those PCs up to a predetermined total percent variance explained, such as, 95%. Figure 6 depicts the prediction performances on colon cancer data, with PCA+LS-SVM(RBF), at different fractions of explained total variance. It shows the results vary with the selected components. Here the number of retained components, depends on the chosen fraction of explained total variance. The proposed approach offers a data-driven selection criterion for PCA problem, instead of a traditional trial and error PC selection.

### Comparison between the proposed criterion and an existing optimization algorithm for RBF-KPCA

**Figure 7:**
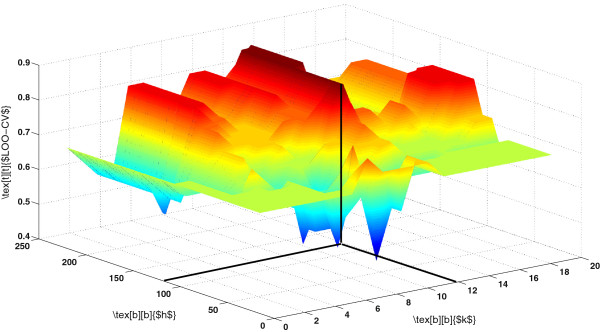
**LOOCV performance of optimization algorithm [**[17]**] on high-grade glioma data set.**

We selected two experiments from Pochet *et al.*[17] (last two data sets in Table 1), being high-grade glioma and breast cancer II data sets. We repeated the same experiments as reported in Pochet *et al.*[17] and compared with the proposed strategy. The results are shown in Table 5. The three dimensional surface plot of LOOCV performance of the method proposed by [17] for the high-grade glioma data set is shown in Figure 7, with the optimal *h*=114.018 and *k*=12. The optimum parameters are *h*=94.868 and *k*=10 obtained by the proposed strategy (see Eq. 6) for the same data set.

**Table 5:** **Comparison of performance of proposed criterion with the method proposed by Pochet *et al. * [**[17]**]: Averaged test AUC(std) over 30 iterations and execution time in minutes**

**Data set**	**Proposed strategy**		**Pochet *et al. *** [17]	
	**Test AUC**	**Time**	**Test AUC**	**Time**
High-grade glioma data	0.746 (0.071)	2	0.704 (0.104)	38
Breast cancer II	0.6747 (0.1057)	4	0.603 (0.157)	459

When looking at test AUC in Table 5, both case studies applying the proposed strategy, perform better than the method proposed by Pochet *et al.*[17] with less variability. In addition, the tuning method Pochet *et al.*[17] appears to be quite time consuming, whereas the proposed model enjoys a distinctive advantage with its low time complexity to carry out the same process.

### Comparison between the proposed criterion and other classifiers

In Table 4, we have highlighted the comparisons in which the proposed method was significantly better. When looking specifically on the performance of each of the discussed methods, we note that LS-SVM performance was slightly low on PCA. On data sets IV, VI, VII proposed approach performs better than, LS-SVM with RBF kernel and LS-SVM with linear kernel. The proposed approach is outperformed, by the t-test + LS-SVM on data sets V and VI and, by both PAM and Lasso on most of the data sets.

## Discussions

The obtained test AUC of different classifiers on nine data sets, do not direct to a common conclusion that one method outperforms the other. Instead, it shows that each of these methods have its own advantage in classification tasks. When considering classification problems without dimensionality reduction, the regularized LS-SVM classifier shows a good performance on 50 percentage of data sets. Up till now, most microarray data sets are smaller in the sense of number of features and samples, but it is expected that these data sets might become larger or perhaps represent more complex classification problems in the future. In this situation, dimensionality reduction processes (feature selection and feature transformation) are the essential steps for building stable, robust and interpretable classifiers on these kind of data.

The selected features of feature selection method such as t-test, PAM and Lasso widely vary for each random iteration. Moreover, the classification performance of these methods on each iteration depends on the number of features selected. Table 6 shows the range, i.e. minimum and maximum number of features selected on 30 iterations. However PAM is a user friendly toolbox for gene selection and classification tasks, its performance depends heavily on the selected features. In addition, it is interesting that the Lasso selected only very small subsets of the actual data sets. But, in the Lasso, the amount of shrinkage varies, depending on the value of the tuning parameter, which is often determined by cross validation [37]. The number of genes selected as the outcome-predictive genes, generally decrease as the value of the tuning parameter increases. The optimal value of the tuning parameter, that maximizes the prediction accuracy is determined; however, the set of genes identified using the optimal value contains the non-outcome-predictive genes (ie, false positive genes) in many cases [9].

**Table 6:** Summary of the range (minimum to maximum) of features selected over 30 iterations

**Data set**	**t-test ( *p *** **<0 *. *** **05)**	**PAM**	**Lasso**
1: Colon	197-323	15-373	8-36
2: Breast	993-1124	13-4718	7-87
3: Pancreatic	2713-4855	3-1514	12-112
4: Cervical	5858-6756	2-10692	5-67
5: Leukemia	1089-2654	137-11453	2-69
6: Ovarian	7341-7841	34-278	62-132
7: Head and neck squamous			
cell carcinoma	307-831	1-12625	3-35
8: Duchenne muscular dystrophy	973-2031	129-22283	8-24
9: HIV encephalitis	941-1422	1-12625	1-20

p-value: False Discovery Rate (FDR) corrected.

The test AUC on all nine case studies shows that KPCA performs better than classical PCA. But the parameters of KPCA need to be optimized. Here we have used LOOCV approach for parameters selection (bandwidth and number of components) of KPCA. In the optimization algorithm proposed by Pochet *et al.*[17], the combination of KPCA with RBF kernel followed by FDA tends to result in overfitting. The proposed parameter selection criterion of KPCA with RBF kernel, often results in test set performances (see Table 4) that is better than using KPCA with a linear kernel, which reported in Pochet *et al.* It means that LOOCV in the proposed parameter selection criterion does not encounter an overfitting for KPCA with RBF kernel function. In addition, the optimization algorithm proposed by Pochet *et al.* is completely coupled with the subsequent classifier and thus it appears to be very time-consuming.

In combination with classification methods, microarray data analysis can be useful to guide clinical management in cancer studies. In this study, several mathematical and statistical techniques were evaluated and compared in order to optimize the performance of clinical predictions based on microarray data. Considering the possibility of increasing size and complexity of microarray data sets in future, dimensionality reduction and nonlinear techniques have its own significance. In many cases, in a specific application context the best feature set is still important (e.g. drug discovery). While considering the stability and performance (both accuracy and execution time) of classifiers, the proposed methodology has its own importance to predict classes, of future samples of known disease cases.

Finally this work could be extended further to uncover key features from biological data sets. In several studies, KPCA have used to obtain biologically relevant features such as genes [38,39] or detect the association between multiple SNPs and disease [40]. In all these cases, one needs to address the parameter optimization of KPCA. The available bandwidth selection techniques of KPCA are time-consuming with high computational burden. This could be resolved with the proposed data-driven bandwidth selection criterion for KPCA.

## Conclusion

The objective in class prediction with microarray data is an accurate classification of cancerous samples, which allows directed and more successful therapies. In this paper, we proposed a new data-driven bandwidth selection criterion for KPCA (which is a well defined preprocessing technique). In particular, we optimize the bandwidth and the number of components by maximizing the projected variance of KPCA. In addition, we compared several data preprocessing techniques prior to classification. In all the case studies, most of these preprocessing steps performed well on classification with approximately similar performance. We observed that in feature selection methods selected features widely vary on each iteration. Hence it is difficult, even impossible to design a stable class predictor for future samples with these methods. Experiments on nine data sets show that the proposed strategy provides a stable preprocessing algorithm for classification of high dimensional data with good performance on test data.

The advantages of the proposed KPCA+LS-SVM classifier were presented in four aspects. First, we propose a data-driven bandwidth selection criterion for KPCA by tuning the optimum bandwidth and the number of principal components. Second, we illustrate that the performance of the proposed strategy is significantly better than an existing optimization algorithm for KPCA. Third, its classification performance is not sensitive to any number of selected genes, so the proposed method is more stable than others proposed in literature. Fourth, it reduces the dimensionality of the data while keeping as much information as possible of the original data. This leads to computationally less expensive and more stable results for massive microarray classification.

## Competing interests

The authors declare that they have no competing interests.

## Authors’ contributions

MT performed bandwidth selection, subsequent classification and drafted the paper. KDB participated in the design and implementation of framework. KDB and BDM helped draft the manuscript. All authors read and approved the final manuscript.
